# Clouds enhance Greenland ice sheet meltwater runoff

**DOI:** 10.1038/ncomms10266

**Published:** 2016-01-12

**Authors:** K. Van Tricht, S. Lhermitte, J. T. M. Lenaerts, I. V. Gorodetskaya, T. S. L'Ecuyer, B. Noël, M. R. van den Broeke, D. D. Turner, N. P. M. van Lipzig

**Affiliations:** 1 KU Leuven, Department of Earth and Environmental Sciences, Celestijnenlaan 200E, Leuven 3001, Belgium; 2 Institute for Marine and Atmospheric research Utrecht—Utrecht University, Utrecht 3584CC, The Netherlands; 3 Atmospheric and Oceanic Sciences, University of Wisconsin–Madison, Madison, Wisconsin 53706, USA; 4 National Severe Storms Laboratory, NOAA, Norman, Oklahoma 73072, USA

## Abstract

The Greenland ice sheet has become one of the main contributors to global sea level rise, predominantly through increased meltwater runoff. The main drivers of Greenland ice sheet runoff, however, remain poorly understood. Here we show that clouds enhance meltwater runoff by about one-third relative to clear skies, using a unique combination of active satellite observations, climate model data and snow model simulations. This impact results from a cloud radiative effect of 29.5 (±5.2) W m^−2^. Contrary to conventional wisdom, however, the Greenland ice sheet responds to this energy through a new pathway by which clouds reduce meltwater refreezing as opposed to increasing surface melt directly, thereby accelerating bare-ice exposure and enhancing meltwater runoff. The high sensitivity of the Greenland ice sheet to both ice-only and liquid-bearing clouds highlights the need for accurate cloud representations in climate models, to better predict future contributions of the Greenland ice sheet to global sea level rise.

## Abstract (web summary)

Clouds play a pivotal role in the energy and mass balance of the Greenland ice sheet, thereby affecting its contribution to global sea-level rise. Here, using a combination of observations and model simulations, the authors show that clouds enhance Greenland ice sheet meltwater runoff by more than 30%.

## Graphical abstract

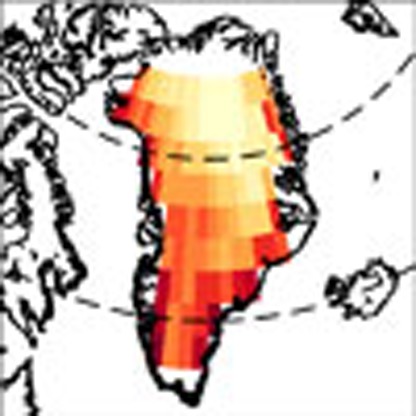

Regionally amplified^1^ anthropogenic climate change^2^ in combination with anomalous large-scale atmospheric circulations^3^ have made the Greenland ice sheet (GrIS) a dominant contributor^4^,^5^ to recent global sea level rise (0.63±0.07 mm per year during 2000–2012; ref. 6). This increasing contribution is primarily driven by a reduction in GrIS surface mass balance (SMB)^6^,^7^,^8^, related to a significant increase in GrIS surface melt extent^9^ and volume^10^. Surface melt is determined by the surface energy balance (SEB)^11^, the sum of incoming and outgoing energy fluxes at the surface.

Clouds are known to play a pivotal role in regulating the local SEB, with competing warming and cooling effects on the surface^12^,^13^,^14^. The dominating effect depends strongly on cloud properties such as vertically integrated ice and liquid water contents that determine cloud optical depth and emissivity, in addition to cloud temperature, sun position and surface albedo^13^,^14^,^15^.

Despite their importance, the distribution of clouds over the GrIS, their phase partitioning (for example, ice-only clouds versus liquid-bearing or mixed-phase clouds) and their precise impact on the SEB and SMB are poorly understood, owing to the limited availability of measurements. Sensitivity studies based on observations have been limited to particular events or cloud types such as the role of low-level liquid-bearing clouds^16^ together with the enhanced poleward transport of humid and warm air^17^ in the July 2012 extreme GrIS melt event, whereas ice sheet-wide assessments of cloud SEB impact relied on climate model output to overcome the limited availability of observations^18^,^19^,^20^.

However, analysis of the recent Coupled Model Intercomparison Project phase 5 model ensemble and a regional climate model shows a significant inter-model spread in the estimates of both ice-only and liquid-bearing cloud frequency and water paths over the GrIS (Supplementary Fig. 1 and Supplementary Methods). This highlights the need for an observation-based assessment of the cloud distribution over the GrIS.

In this study, we use a unique combination of state-of-the-art satellite remote sensing, ground-based observations and a regional climate model, to quantify the impact of clouds on the SEB over the entire GrIS. The GrIS shows a strong sensitivity to cloud presence, where both ice-only and liquid-bearing clouds are warming the ice sheet surface. Using an advanced snow model, we find that this warming enhances GrIS meltwater runoff in response to reduced refreezing rates at night, when cloud warming is highest compared with clear skies.

## Results

### Cloud properties from active satellite remote sensing

In this study, a total of ∼6.3 million satellite cloud observations were compiled over the GrIS for the period 2007–2010 from the CloudSat and CALIPSO (Cloud-Aerosol Lidar and Infrared Pathfinder Satellite Observation) satellites. These missions carry the first complementary collocated spaceborne radar and lidar sensors^21^,^22^, providing information on vertical distributions of cloud ice and liquid water contents, cloud phase and effective radii, supplemented by cloud optical depths derived from MODerate resolution Imaging Spectroradiometer (MODIS)^23^.

These observation-based vertical profiles of cloud properties provide the main input for radiative flux calculations in the level-2 ‘Fluxes and Heating Rates' product (2B-FLXHR-LIDAR)^24^. An updated refined version of the 2B-FLXHR-LIDAR algorithm that includes low-level supercooled liquid-bearing clouds detected using a combination of CloudSat and CALIPSO observations^25^ is used in this study. This new data set, which will form the basis of the Release 05 version of the CloudSat 2B-FLXHR-LIDAR product, provides for the first time a robust partitioning of ice-only and liquid-bearing clouds that does not suffer from errors typically present in conventional satellite products^25^,^26^, and provides an unprecedented three-dimensional view of clouds over the GrIS and their microphysical properties^22^.

**Figure 1:**
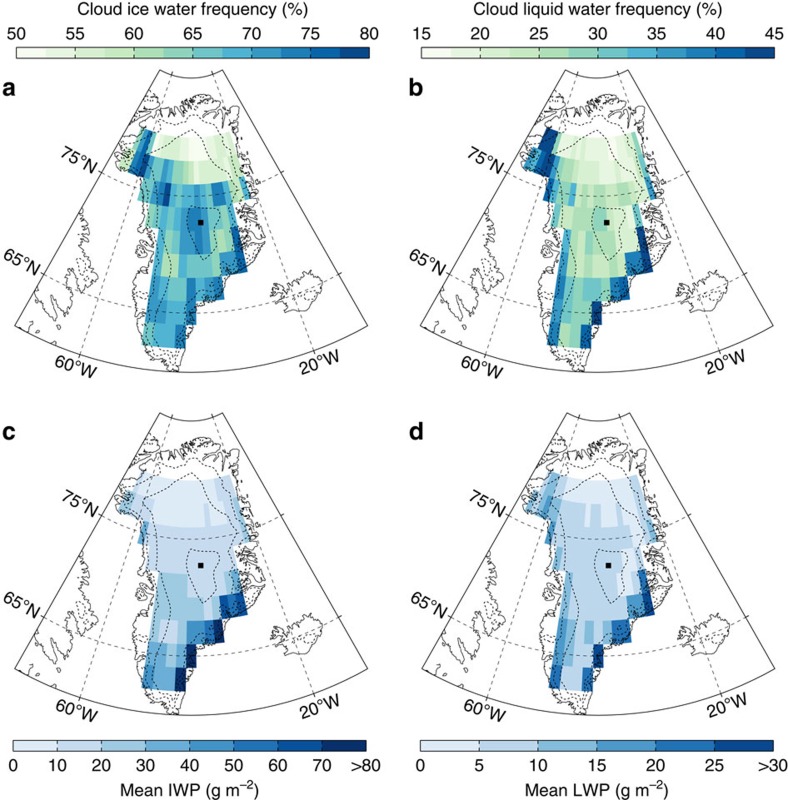
Retrieved cloud properties over the GrIS by satellite remote sensing. These retrievals result from joint CloudSat, CALIPSO and MODIS observations. Both (**a**) cloud ice and (**b**) cloud liquid water occur frequently over the GrIS, although strong spatial variations exist. (**c**) Mean IWP in all conditions and (**d**) mean LWP in all conditions also show spatial variability in cloud water contents. Dashed curves indicate 1,000 m height contours and the black dot represents the location of the Summit station.

Figure 1 shows that clouds occur frequently over the GrIS, where the annual mean cloud cover is 67% (Table 1). The CloudSat and CALIPSO data sets employed here suggest that liquid-bearing clouds that contain both ice and (supercooled) liquid water are present 28% of the time, consistent with other work showing that such clouds are prevalent throughout the Arctic^27^. There were almost no (<1%) detections of liquid-only clouds over the GrIS. Whereas the average total cloud fraction is fairly stable throughout the year ranging from 63 to 70%, the satellites observe strong spatial (Fig. 1) and seasonal (Table 1) variations in ice-water path (IWP), liquid-water path (LWP) and liquid/ice partitioning. Liquid-bearing clouds occur predominantly during summer (46% of the time), associated with the presence of warmer and moister air^28^, whereas winter is characterized by a much higher frequency of ice-only clouds (55%). Ice clouds occur frequently over the entire GrIS, whereas liquid-bearing clouds are much more abundant in the coastal regions. Annual mean cloud LWP in all conditions varies between 3 and 33 g m^−2^ over the entire GrIS with highest values in the coastal zones, whereas IWP is lowest (<7 g m^−2^) in the northern part of the interior and highest (>100 g m^−2^) in the high-snowfall coastal zones of the southeastern GrIS.

### Cloud radiative effect

The satellite-based cloud observations allow to estimate the cloud impact on the SEB using a two-stream broadband radiative transfer model (see Methods). Cloud impact is defined here as the net cloud radiative effect (CRE)^5^ at the surface, which is the difference between the all-sky and clear-sky surface radiation budgets^12^.

**Figure 2:**
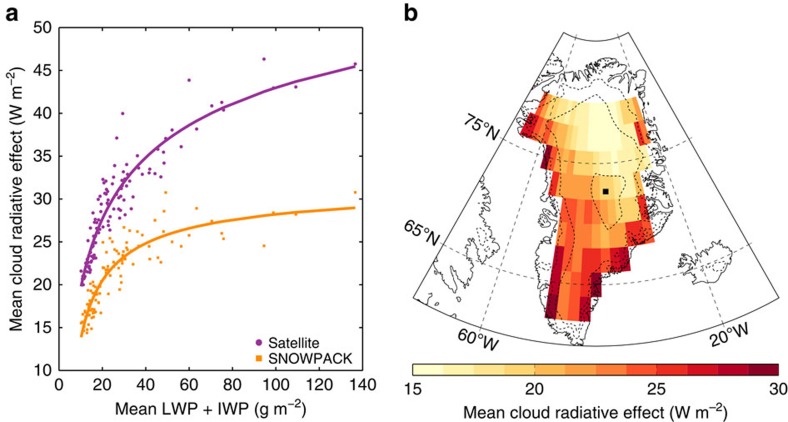
CRE over the GrIS. (**a**) The average relationship between mean LWP+IWP and mean annual CRE is positive at each location, with higher radiative effects from optically thicker (more liquid and/or ice) or more frequent clouds. The purple circles were derived from satellite observations, whereas the orange squares result from the SNOWPACK runs. The satellite-retrieved CRE estimates assume fixed surface conditions (albedo and surface temperature). In reality, these conditions may change in the absence of the detected clouds. The entire snowpack changes as a result of cloud warming, reducing the initial CRE (lower values in the orange curve). (**b**) Yearly mean CRE over the GrIS (2007–2010) from the SNOWPACK output. This estimate takes into account changing surface conditions (albedo and surface temperature) and snowpack with changing energy inputs due to the presence/absense of clouds. Dashed lines indicate 1,000 m height contours and the black dot represents the location of the Summit station.

The spatial distribution of the annual mean CRE is positive over the entire GrIS and is strongly correlated with LWP and IWP (Fig. 2a). This indicates that longwave (LW) warming dominates shortwave (SW) cooling, which can be explained by the high solar zenith angles (SZAs) and surface albedo^29^.

Averaged over the GrIS, clouds reduce annual mean surface radiative heat loss by 29.5 (±5.2) W m^−2^, a factor of 2.7 decrease relative to clear skies. Cloud liquid water contributes only 11.2 (±2.4) W m^−2^ to the total radiative effect. This is explained by the lower occurrence frequency and strong seasonal cycle of liquid-bearing clouds with highest frequencies in summer, when both LW warming and SW cooling constitute the CRE. Ice-only clouds occur most frequently in winter, when only LW warming contributes to the radiative effect.

### Translating SEB to SMB using a snow model

The annual mean CRE of 29.5 W m^−2^ provides enough energy to melt ∼90 Gt of ice in the GrIS ablation area during July and August. However, part of this excess energy is absorbed by the snowpack, warming the surface and enhancing outgoing LW radiation. Warmer snow promotes snow metamorphism, which lowers the albedo of the snowpack^30^, whereas multiple reflections of SW radiation between clouds and the surface in combination with spectrally dependent cloud absorption generally increase it^31^,^32^.

To account for these processes, which are not all included in the satellite product, the one-dimensional physical snow model SNOWPACK^33^ was used. The cloud impact on the GrIS SMB is assessed by forcing SNOWPACK with different cloud inputs (all-sky, clear-sky and no-liquid). As our SNOWPACK simulations require hourly meteorological input data, which is much more frequent than the satellite overpasses (on average CloudSat and CALIPSO observe each 2° by 2° grid box once every 1–7 days, depending on the location), we constructed a hybrid satellite-climate model data set, combining the benefits of the observationally based estimates of CREs with the hourly temporal resolution of a regional climate model (see Methods).

**Figure 3:**
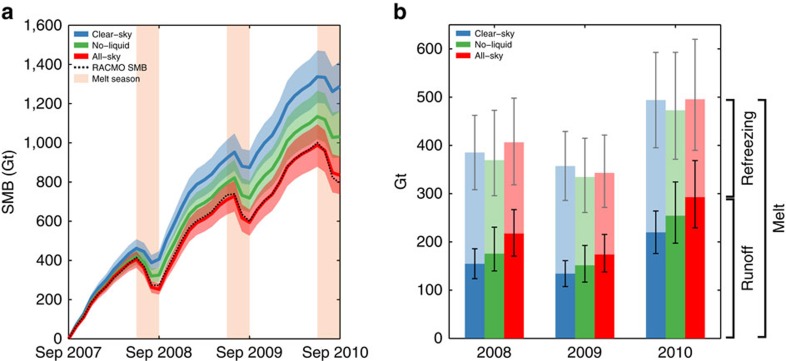
The effects of clouds on the SMB during the period September 2007–September 2010. (**a**) Evolution of GrIS SMB indicates that the cloudy simulations have a lower SMB. Uncertainties are shown by the shaded areas. The black curve represents the regional climate model RACMO2.3 SMB values and show the performance of SNOWPACK in simulating the GrIS SMB. (**b**) Yearly GrIS melt, refreezing and runoff. Despite negligible differences in melt, 58% of the meltwater refreezes in clear-sky conditions, whereas only 45% refreezes in all-sky conditions. Annual meltwater runoff is therefore about one-third higher in the presence of clouds, with a slightly higher contribution of liquid-bearing clouds. The whiskers indicate an inherent SNOWPACK uncertainty and the sensitivity to the amount of LWP/IWP in the SNOWPACK simulations.

The snow model simulations, which capture the evolution of the GrIS SMB from 2007 to 2010 (Fig. 3a, black dashed curve), indicate that clouds warm the GrIS surface by 1.2 (±0.1) °C on average over the entire period. The changes in the snowpack as a result of this cloud-induced warming, however, in combination with increased albedo under cloudy conditions, lower the annual mean CRE (Fig. 2a, orange line) by 8.4 W m^−2^ (∼30%) relative to the satellite estimate (Fig. 2a, purple line). The spatial distribution of this updated CRE is shown in Fig. 2b.

### Enhanced meltwater runoff

Although GrIS surface melt is nearly identical under all-sky and clear-sky conditions (Fig. 3b), clouds enhance GrIS meltwater runoff by 56±20 Gt per year (32±12 %), with similar contributions from cloud ice (25 Gt) and cloud liquid (31 Gt) water. This suggests that the primary influence of clouds is by reducing meltwater refreezing; in clear-sky conditions, about 58% of the meltwater refreezes, but this fraction decreases to 45% in the presence of clouds.

**Figure 4:**
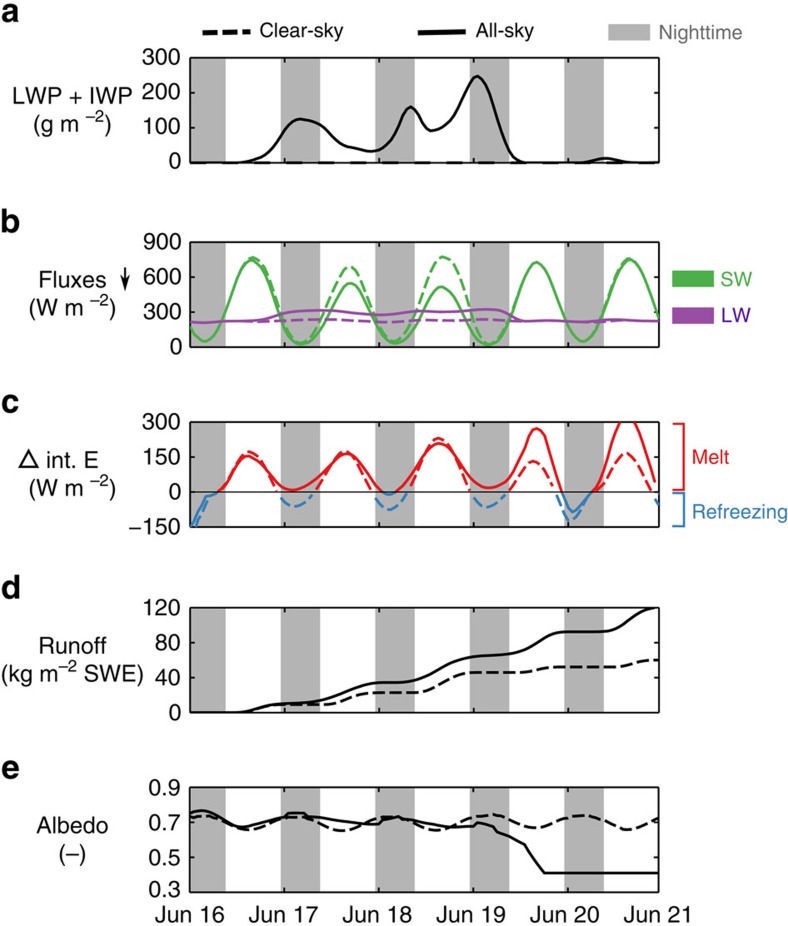
Case study showing cloud impacts on melt/refreezing during 16–21 June 2008 at 67°N–49°E. Solid curves represent all-sky conditions and dashed curves represent clear-sky conditions. Grey zones are characterized by a SZA>70°, considered to be nighttime. All variables as shown in the figure have been smoothed using a 6-hourly moving-average window. (**a**) Total (liquid+ice) water path, showing a cloud in the all-sky simulation. (**b**) Downwelling SW (green) and LW (purple) radiative fluxes. In the presence of a cloud, SW cooling (solid curve below dashed curve) and LW warming (solid curve above dashed curve) occur simultaneously during daytime, whereas LW warming dominates nighttime. (**c**) Internal energy change of the snowpack due to melting (positive) and refreezing (negative) processes. Melt rates are highest at solar noon when SW insolation peaks, whereas refreezing rates are highest at night when strong surface radiative cooling dominates. In the presence of clouds, this radiative cooling is reduced, impeding the refreezing mechanism. (**d**) Cumulative meltwater runoff in snow water equivalent (SWE) is higher under cloudy conditions, due to limited meltwater refreezing and earlier bare-ice exposure. (**e**) Surface albedo as simulated by SNOWPACK. Persistent warming by clouds enhances meltwater runoff, leading to an earlier exposure of bare ice and slush that have a much lower albedo than (fresh) snow (from 19 June onwards). At this point, the warming is amplified due to a much higher absorption of SW radiation in the all-sky simulation, as opposed to the clear-sky simulation.

The governing mechanisms that explain this effect are illustrated in Fig. 4, for a case study during 16–21 June 2008 at 67°N-49°E (ablation zone), during an albedo transition period. In the presence of a thick cloud (Fig. 4a), strong SW cooling and LW warming partly compensate during daytime (Fig. 4b). Surface melt occurs during and shortly after solar noon, when incoming SW radiation peaks (Fig. 4c, red curves). During the subsequent nighttime hours, when incoming SW is small (Fig. 4b, grey zones), clear-sky conditions favour strong surface radiative cooling, extracting heat from the warmer subsurface snow by conduction, (partly) refreezing the meltwater (Fig. 4c, blue curves). This radiative cooling is reduced under cloudy conditions, impeding the refreezing mechanism. The additional meltwater that does not refreeze fills up the available pore space until it runs off^34^ (Fig. 4d), whereas a persistent positive CRE leads to firn warming, further enhancing melt and subsequent runoff^35^. The combined effect causes an earlier exposure of bare ice and slush under cloudy conditions, which amplifies these mechanisms through enhanced SW absorption with lower albedo values (albedo feedback, Fig. 4e). During the last 2 days of the case study, when the cloud has disappeared in the all-sky simulation, this albedo feedback is strengthened due to increased SW insolation in the absence of cloud SW blocking, enhancing melt relative to the clear-sky simulation (Fig. 4c, red curves).

Along with meltwater runoff, our results suggest that clouds enhance GrIS sublimation. In summary, liquid-bearing and ice-only clouds contribute equally to a GrIS SMB decrease during 2007–2010 of 35±5% relative to clear-sky conditions by increasing meltwater runoff and sublimation, which is the average equivalent of 152±20 Gt per year (Fig. 3a, red curve).

## Discussion

Our results highlight the strong sensitivity of the GrIS SEB and SMB to clouds. This analysis overcomes many of the current issues that exist in observing and modelling of mixed-phase clouds by creating a hybrid satellite-climate model data set that closely matches satellite retrievals and ground-based observations with hourly temporal resolution. The strong positive CRE over the GrIS is found to warm the surface, enhance meltwater runoff in the GrIS ablation area and increase sublimation in the GrIS accumulation area.

However, a substantial uncertainty is associated to the sublimation term, as SNOWPACK was used in an offline configuration and does not account for any feedbacks to the atmosphere in response to changes in the snowpack. In reality, changes in surface temperature induced by clouds will modify turbulent fluxes and thus the boundary-layer air temperature and humidity. Here we did not include these second-order effects and focused exclusively on the sensitivity of the GrIS to cloud-related changes in the SEB. This constitutes a first important step in assessing the impact of clouds on the GrIS SMB.

These results further indicate that not only liquid-bearing clouds^16^ but also clouds composed exclusively of ice significantly increase radiative fluxes into the surface and decrease GrIS SMB. This underscores the need for continued research into the factors that govern the formation and maintenance of these distinct cloud regimes, and their evolution in a future warmer and wetter Arctic^36^. Evidence of the large spread in cloud cover and liquid/ice partitioning over the GrIS in current state-of-the-art climate models, in combination with our limited understanding of the interaction between clouds, circulation and climate^37^, suggests that improved cloud representations in climate models could significantly increase the fidelity of future projections of GrIS SMB and subsequent global sea level rise.

## Methods

### General approach

State-of-the-art satellite cloud observations have been used in a refined multi-stage hybrid model-observation algorithm, to estimate surface radiative fluxes. To overcome the limited temporal resolution of these satellite observations, a regional climate model has been incorporated to interpolate the observations to hourly resolution. Ground-based observations from Summit^28^,^38^ and several automatic weather stations (AWSs) have been used to evaluate the resulting hybrid satellite-climate model data set. This new hybrid data set was finally used to force a snow model that simulates the impact of the CRE on the GrIS SMB.

### CloudSat/CALIPSO satellite observations

Although radiative fluxes over the GrIS are available from the European Centre for Medium-Range Weather Forecasts (ECMWF) atmospheric re-analysis (ERA), the uncertainty in these fluxes is large, as they are calculated based on modelled clouds, excluding any cloud observational data^39^. However, with the launch of the CloudSat and CALIPSO satellites, radiative flux calculations can now be constrained by a comprehensive set of cloud observations from space^21^,^22^,^23^. The Cloud-Aerosol Lidar with Orthogonal Polarization (CALIOP) sensor aboard CALIPSO was designed to focus on optically thin clouds and the 94-GHz Cloud Profiling Radar (CPR) aboard CloudSat probes optically thicker clouds and precipitation. CALIOP and CPR measure the backscattered energy by cloud particles, which can be converted into vertical distributions of cloud ice and liquid water contents, and effective radii, filled in by MODIS radiance information when the retrieval algorithms of the active sensors fail to converge. Despite their limited spatiotemporal sampling along specific tracks and limited collocated availability from 2007 to 2010, these active satellite cloud observations have been proven to be of great relevance for polar cloud studies^39^,^40^.

### The 2B-FLXHR-LIDAR algorithm

The primary satellite product used in this study is the refined CloudSat 2B-FLXHR-LIDAR^24^. This product combines (i) cloud and aerosol observations from CloudSat, CALIPSO and MODIS; (ii) ancillary temperature and humidity profiles from ECMWF atmospheric re-analyses; and (iii) International Geosphere-Biosphere Programme surface albedo/emissivity data to constrain the two-stream radiative transfer model (RTM) BugsRad^24^, which calculates broadband radiative fluxes. The CloudSat footprint of 1.4 km × 1.8 km provides the basis for the radiative transfer calculations, determining the horizontal resolution of the product, whereas the vertical resolution is 240 m.

We found that in the presence of low-level supercooled liquid-bearing clouds that occur frequently over the Arctic^27^, the original 2B-FLXHR-LIDAR product was severely underestimating downwelling LW fluxes because of the inability of the existing algorithm to properly handle these clouds, which were falsely identified as ice-only clouds. This was attributed to the fact that the CloudSat CPR is insensitive to small supercooled liquid droplets, while an exact retrieval of water path in this case was often not available from the CALIOP sensor. In such an event, a simple linear temperature-based relationship was used in the original version, to decide on the portion of ice and liquid water to which the CALIOP backscattered energy was translated, not allowing any supercooled liquid water to occur below −20 °C, while it can physically occur down to −40 °C. In this study, we therefore refined the 2B-FLXHR-LIDAR product by introducing the use of the CloudSat level-2 ‘Combined Radar and Lidar Cloud Scenario Classification' product^25^,^41^ as input for the refined 2B-FLXHR-LIDAR algorithm, which combines information from the CloudSat CPR and CALIPSO CALIOP to identify cloud phase. When low-level supercooled liquid-bearing clouds are detected and no exact retrieval is available for determining the LWP that is assigned to these clouds, we rely on a fill value based on the median LWP value retrieved by ground-based observations at Summit (see below).

A total of ∼6.3 million refined 2B-FLXHR-LIDAR flux profiles were aggregated in a 2° by 2° grid, to ensure a sufficient amount of satellite profiles within each grid box. Ice and liquid water occurrence frequencies and water paths were extracted from the refined 2B-FLXHR-LIDAR product and represent the combined CloudSat/CALIPSO/MODIS observations that were used in the radiative flux retrievals.

The radiative fluxes of the original 2B-FLXHR-LIDAR product have been evaluated^24^ through comparisons with Clouds and the Earth's Radiant Energy System (CERES) top-of-atmosphere observations and surface flux retrievals on a global scale. To assess the quality of the refined 2B-FLXHR-LIDAR product over the GrIS, the satellite LWP retrievals were first evaluated with ground-based LWP measurements^42^, retrieved by a microwave radiometer (MWR) installed at Summit, Greenland, as part of the ICECAPS project^28^, during July 2010–December 2010. Despite the limited available overlap in time of 6 months, relative frequency histograms of LWP show a strong agreement between MWR-retrieved and satellite-retrieved LWP values (Supplementary Fig. 2). Furthermore, cloud macrophysical properties such as cloud fraction agree well (not shown) with the available ground-based observations^28^,^38^.

An additional evaluation was performed by comparing the retrieved downwelling LW/SW radiative fluxes from the satellite product with ground-based flux measurements from 11 AWSs on the GrIS (IMAU^29^ and PROMICE, http://www.promice.org/). Monthly averages were computed for satellite overpasses within 100 km from the AWS locations, whereas for consistency with the original overpasses, monthly averages for the AWS-measured fluxes were based on interpolated flux measurements to the times of these overpasses. AWS stations that are located in the same 2° by 2° grid box were averaged to yield one representative flux observation for that grid box. The resulting eight comparisons (Supplementary Fig. 3) show the good performance of the 2B-FLXHR-LIDAR surface radiative fluxes, as there is in general a close agreement between satellite-retrieved and AWS-measured LW and SW fluxes. Statistics on bias and root-mean-square errors (RMSEs) are given in Supplementary Table 1. Although there is a considerable range in these statistics, the overall agreement is good, with a mean bias of −4 and 7.6 W m^−2^ for downwelling LW and SW, respectively, well below the estimated monthly mean measurement uncertainty of ∼5% in the radiation instruments^29^,^43^. RMSEs equal 13.2 W m^−2^ for LW fluxes and 24.2 W m^−2^ for SW fluxes; these RMSE values are very similar to radiative flux closure results shown at Barrow, Alaska, where the cloud properties were specified by ground-based radars, lidars and radiometers^44^. Local disagreement between satellite and AWS can be explained by heterogeneous terrain within the 100-km radius of the AWS, AWS sensor uncertainties, AWS sensor tilt affecting SW measurements and low sampling rate for some AWS locations to compare 2B-FLXHR-LIDAR and AWS observations. Despite this, our evaluation indicates that the refined 2B-FLXHR-LIDAR product provides reliable estimates of radiative fluxes over the GrIS.

### Cloud radiative effect

CRE at the surface (W m^−2^) is defined by equation (1), where 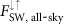 and 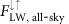 are the net surface radiative SW and LW fluxes in all-sky conditions, and 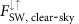 and 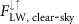 are the net surface radiative fluxes that would occur in the absence of clouds^12^.


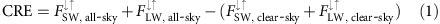


A positive effect indicates net cloud warming at the surface, whereas a negative effect is indicative of net cloud cooling. The magnitude of the radiative effect is intimately connected to the amount of ice and liquid water in the cloud, its temperature, SZA and surface albedo^14^.

All analyses of CRE over the GrIS were focused on three different cloud scenarios. In the ‘all-sky' simulation (that is, control run), radiative fluxes were calculated based on the satellite-observed clouds and their LWP and IWP. In the ‘clear-sky' simulation, all cloud ice and liquid water was removed (that is, IWP and LWP were set to zero), to simulate the radiative fluxes that would occur in the absence of clouds. In the ‘no-liquid' simulation, only the retrieved cloud liquid water was removed (LWP set to zero), effectively eliminating the impact of (supercooled) liquid water in liquid-bearing clouds on the calculated radiative fluxes, whereas IWP was kept unchanged. All-sky CRE is defined as the difference in radiative fluxes between the ‘all-sky' and the ‘clear-sky' simulations, whereas liquid-bearing CRE is defined as the difference in radiative fluxes between the ‘all-sky' and the ‘no-liquid' simulations.

To determine an observationally based CRE estimate, the 2B-FLXHR-LIDAR RTM was used for the three different cloud scenarios described above. Because of the large computational requirements of running the RTM on all satellite observations between 2007 and 2010, the ‘clear-sky' and ‘no-liquid' simulations were limited to 4 months, distributed over the year 2010 to be able to capture seasonal variability. For this purpose, all available observations from March, June, September and December 2010 were used.

Downwelling LW radiation is described by equation (2)^45^, in which LW_as_ ↓ and LW_cs_ ↓ are the downwelling LW radiation in all-sky and clear-sky conditions, respectively, *ɛ*_cs_ is the clear-sky atmospheric emissivity, *T* is the temperature (K) at a reference height, *σ* is the Stefan–Boltzmann constant and *F* is the cloud enhancement factor.


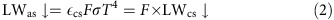


The cloud enhancement factor *F* (⩾1) describes the increase in downwelling LW radiation relative to clear sky due to clouds^46^. In this study we estimate *F* for all clouds and for liquid water in liquid-bearing clouds using the previously described RTM runs, in which respectively all clouds (‘clear-sky' simulation) and liquid water in liquid-bearing clouds (‘no-liquid' simulation) were removed from the satellite profiles, while keeping all other parameters unchanged.

Subsequently, *F*_all, LW_ and *F*_liq, LW_, that is, the LW cloud enhancement factors *F* for all clouds and liquid-bearing clouds, respectively, can be calculated by equations (3) and (4).


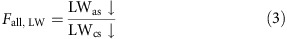



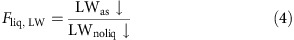


CRE in the LW, and therefore the LW cloud enhancement factor, is primarily a function of cloud temperature (often approximated by cloud-base temperature (CBT)), cloud height and emissivity^14^. The latter is mainly determined by the cloud LWP and IWP.

In an analogous manner, we can define an SW cloud enhancement factor that describes the decrease in downwelling SW radiation due to clouds relative to clear-sky conditions. Similar to equations (3) and (4), cloud enhancement factors *F*_all, SW_ and *F*_liq, SW_ (≤1) that account for the decrease in SW radiation due to all clouds and liquid-bearing clouds, respectively, can be defined by equations (5) and (6).


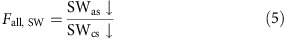



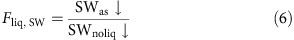


The SW cloud enhancement factor mainly depends on cloud microphysical properties (LWP and IWP), surface albedo and SZA^13^.

To extend the satellite CRE data set from the four available months to the full 2007–2010 period, we trained neural networks to predict *F*_all, LW_, *F*_all, SW_, *F*_liq, LW_ and *F*_liq, SW_ (ref. 47).

Inputs for the neural networks to predict the cloud enhancement factors were LWP, IWP and near-surface temperature for LW and LWP, IWP, SZA and surface albedo for SW. LWP, IWP, SZA and surface albedo are readily available in our data sets. However, probably the best temperature-related predictor of LW CRE is cloud temperature or CBT. Yet, this variable is not readily available from the ERA-Interim product used by 2B-FLXHR-LIDAR (‘ECMWF-AUX') and it is not available in the RACMO model outputs that were used in a later stage (see below). Therefore, near-surface temperature (2 m), originating from ECMWF ERA-Interim reanalysis data, was used instead of CBT, to train the neural networks. As such, the method only requires a good correlation between near-surface temperature and CBT. We therefore investigated the relationship between both temperatures, with CBT approximated here by the temperature of the satellite bin containing the cloud base (as detected by the combined CloudSat/CALIPSO observations), for the 4 months explicitly calculated by the RTM, and found a high correlation (*r*=0.83) for cases over the GrIS with LWP+IWP ⩾10 g m^−2^. From this we expect the near-surface temperature to be a good proxy to predict LW CRE.

Hence, LWP, IWP, near-surface temperature, SZA and surface albedo were used as inputs for the neural networks and the cloud enhancement factors *F* as targets. Of the available satellite-retrieved CRE estimates, 70% was used for training the neural networks, whereas 15% was employed to avoid overfitting and 15% was used as independent validation set to assess the neural network performance. As the range of albedo values that are used in the 2B-FLXHR-LIDAR algorithm is relatively small, we performed additional offline RTM simulations with a variable surface albedo, to extend the training data set for a wider range of albedo values. The neural networks for both SW and LW consist of one hidden layer with ten neurons. Supplementary Fig. 4 shows the error histograms of the neural networks, expressed as the difference between the *F* factors predicted by the neural networks, and the *F* factors calculated from the RTM runs. For all *F* factors, biases are small (0±0.03 for *F*_liq, SW_, 0±0.07 for *F*_liq, LW_, 0±0.03 for *F*_all, SW_ and 0±0.05 for *F*_all, LW_), confirming the good skill of the neural networks to predict CRE. The errors on the *F* factors were used to calculate the uncertainty on the estimated CRE over the GrIS (see ‘Uncertainty derivations').

The final all-year CRE as estimated by the neural networks, agrees very well with ground-based observations atop the GrIS at Summit^48^ (30 versus 33 W m^−2^), whereas in the ablation zone of the GrIS this estimate is substantially higher (33 versus 23 W m^−2^), based on AWS stations S6 and S9 (ref. 29). This disparity is almost exclusively attributed to a difference in LW CRE, most probably due to the absence of cloud observations in the ground-based analysis and related assumptions for cloud optical depth retrievals^29^. In addition, we have calculated the monthly mean CRE over 2007–2010 for Summit, to ensure that the seasonal cycle of CRE is captured well. The seasonal pattern shown in Supplementary Fig. 5 agrees well with the results of a previous study^48^, where their retrieval uncertainty spans the observed differences with our results. One clear difference that stands out is a lower LW CRE in our numbers compared with theirs in wintertime. Two possible explanations are (i) the difference in years that are used for the sampling and (ii) the ground-based instruments that are more sensitive to very small amounts of LWP/IWP that are missed in the CloudSat/CALIPSO estimate. Overall, the satellite-based estimates of CRE agree well with ground-based observations.

### Regional climate model RACMO2.3

As the temporal resolution of satellite observations is limited by the amount of overpasses, a hybrid approach was designed (see below) to combine the observational constraints of the satellite data with the temporal resolution of the regional climate model RACMO2.3.

RACMO version 2.3 (ref. 49) combines the atmospheric dynamics from HIRLAM with the physical processes described by ECMWF Integrated Forecast System Cycle 33R1 (ref. 50). RACMO2.3 is specifically adapted for use over polar ice sheets, as it is interactively coupled to a multi-layer snow model^51^, which includes an advanced snow albedo scheme^52^,^53^, accounts for drifting snow^54^ and contains several improvements in the representation of atmospheric physics compared with RACMO2.1. In particular, changes were introduced in the cloud scheme, which now includes a new ice super-saturation parameterization, improving the representation of supercooled liquid-bearing clouds, and also in the turbulence and radiation parameterization schemes^55^,^56^. The original RACMO data were aggregated in the same 2° by 2° grid that was used for the satellite observations.

Part of the results in this study strongly depend on the performance of the RACMO2.3 model. As this study focuses in particular on GrIS melt, it is of utmost importance that the climate is well simulated for reproducing melt events. The RACMO2.3 regional climate model has been extensively evaluated for simulating the near-surface climate, showing a very good performance in representing air temperature and specific and relative humidity^51^,^57^, wind speed^58^,^59^ and surface albedo^52^. With regard to SEB, however, significant biases remain in modelled surface radiative fluxes, with, in particular, an underestimation of the downwelling LW radiation and an overestimation of the downwelling SW radiation, due to an underestimated cloud optical depth^49^. These findings are confirmed in our comparisons between the original RACMO2.3-modelled radiative fluxes and retrieved radiative fluxes by the 2B-FLXHR-LIDAR algorithm (red dots in Supplementary Fig. 6). Downwelling LW radiation is systematically underestimated with a mean bias of −6.2 W m^−2^ and an RMSE value of 8.8 W m^−2^, whereas downwelling SW radiation is slightly overestimated with a mean bias of 2.1 W m^−2^ and an RMSE value of 5.5 W m^−2^. The earlier hypothesis^49^ is supported by our IWP/LWP comparisons in the Supplementary Methods. To improve the modelled IWP/LWP values and reduce the corresponding downwelling SW/LW biases, we therefore constructed a hybrid satellite-climate model data set.

### Hybrid satellite-climate model data set

The accuracy of the satellite observations and high temporal resolution of RACMO2.3 were combined by fitting 3-day moving averages through both the satellite and model LWP/IWP values and calculating an additive correction factor *CF* (that is, the difference between the satellite and model moving average value) at each model output timestep for each 2° by 2° grid box. The *CF* factor is exponentially weighted with respect to the original model values, to avoid that too much LWP/IWP is added when originally no cloud was present in the model. The resulting exponentially weighted factor was used to rescale the LWP/IWP model values, whereas the original model values were retained when no satellite retrieval was available within the moving average window. The correction procedure is mathematically shown in equation (7) for the example of LWP. The factor *P* determines the shape of the exponential function. Both the width of the moving average window and the exponential factor *P* were chosen in such a way that the resulting corrected radiative fluxes resemble the satellite-retrieved radiative fluxes as closely as possible (see below). The factor *P* ranges from 0.03 in summer to 1,000 in winter. The resulting differences between corrected and original IWP values range from −69 to 203 g m^−2^, whereas the difference in LWP values range from −13 to 82 g m^−2^ (99 percentiles).


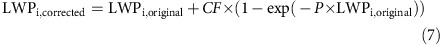


There are no significant lags in time and place for RACMO2.3 simulations compared with satellite observations. RACMO2.3 is forced by ERA-Interim, giving very good correlations with observations for daily and subdaily variations in near-surface climate (temperature, wind speed and humidity)^51^,^57^. We also investigated the possible occurrence of lags in time by performing cross-correlation tests between RACMO2.3 LWP/IWP simulations and the satellite LWP/IWP retrievals, and found no significant lag (not shown).

Comparisons of the original RACMO and hybrid satellite-climate model LWP histograms with the ground-based retrievals by the MWR at Summit are shown in Supplementary Fig. 7. These histograms confirm that the hybrid LWP values agree significantly better with ground-based observations, although LWP values remain somewhat underestimated. Moreover, for the five available months (July to December 2010) with concurrent ground-based observations, the difference in median LWP value has decreased from an underestimation of 5.3 to 1.7 g m^−2^.

After constraining the original model LWP/IWP by satellite observations, the trained neural networks were used to scale the modelled radiative fluxes accordingly. This was done by first converting the original RACMO fluxes to clear-sky fluxes and subsequently converting these clear-sky fluxes to radiative fluxes that match the hybrid LWP/IWP data set. In this framework, clear-sky radiative fluxes were calculated by obtaining cloud enhancement factors *F*_all, LW, original_ and *F*_all, SW, original_ from the neural networks with the original LWP/IWP/T2m/SZA/albedo model values as inputs (equation (8) for the LW example).


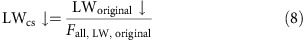


Next, a new set of cloud enhancement factors *F*_all, LW, hybrid_ and *F*_all, SW, hybrid_ was calculated by using the hybrid LWP/IWP data set as inputs for the neural networks, in addition to updated albedo values based on a physical albedo parameterization^60^, which match the corrected optical depths, as albedo generally increases with optical depth^32^. Finally, these adjusted cloud enhancement factors were used to calculate the hybrid all-sky LW_hybrid_ ↓/SW_hybrid_ ↓ fluxes, as shown in equation (9) for the LW.


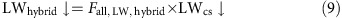


The LWP/IWP correction parameters were chosen in such a way that the corrected downwelling LW/SW fluxes over the entire GrIS match the satellite-retrieved radiative fluxes as closely as possible (Supplementary Fig. 6, blue squares). This reduced the original downwelling LW bias of −6.2 W m^−2^ and RMSE of 8.8 W m^−2^ to −0.9 W m^−2^ and 6.1 W m^−2^, respectively. Downwelling SW bias and RMSE were reduced from 2.1 and 5.5 W m^−2^ to −0.3 and 3.7 W m^−2^, respectively.

Finally, we independently evaluated both the original and hybrid downwelling LW/SW fluxes in RACMO by comparisons with 11 AWSs on the GrIS. The results in Supplementary Fig. 8 show that the systematic underestimation of downwelling LW fluxes at the surface in the original RACMO2.3 model has been greatly reduced in the hybrid satellite-climate model data set. Mean bias in the LW fluxes is reduced from −9.9 to −3.2 W m^−2^, with a reduced RMSE from 10.9 to 6.8 W m^−2^. Mean bias in the SW fluxes is reduced from 4.4 to 1.8 W m^−2^, with no change in RMSE.

CRE estimates for all clouds in the hybrid satellite-climate model data set follow directly from the previous results, whereas an additional set of *F*_liq, LW, hybrid_ and *F*_liq, SW, hybrid_ factors was calculated for estimating CRE by cloud liquid water in liquid-bearing clouds. Surface albedo values from RACMO2.3 were updated for this purpose to include the effect of changing cloud optical depth on the albedo values, following a physical albedo parameterization^60^.

### Simulating the GrIS SMB using SNOWPACK

To couple the effect of clouds on the SEB to the SMB, SNOWPACK simulations were performed for the ‘all-sky', ‘no-liquid' and ‘clear-sky' scenarios on the 2° by 2° grid for the three hydrological years from September 2007 to September 2010, after a 10-year spinup. SNOWPACK^33^ is a one-dimensional physical snow model, which, driven by time series of standard meteorological observations, models the stratigraphy, snow micro-structure, snow metamorphism, snow temperature profile and settlement, as well as surface energy exchange and mass balance of a snowpack. SNOWPACK can be run with a variety of schemes for albedo, metamorphism and water balance. The output of the model is a set of time series that describe the snow profile (albedo, temperature, grain size, density and water content) and its processes (refreezing, water retention and so on). SNOWPACK was developed for seasonal snow, but it has recently been successfully applied to Antarctica as well^61^.

The all-sky simulation (that is, control run) was forced by the hybrid satellite-climate model radiative fluxes and RACMO2.3 meteorological input data (2 m air temperature, 10 m wind speed, 2 m relative humidity and total precipitation), linearly interpolated to hourly values, and represents the most realistic estimate of the actual GrIS SMB. In the ‘clear-sky' simulation the LW and SW CRE of all clouds was removed to simulate the cloud impact (both ice-only and liquid-bearing) on the SMB, whereas in the ‘no-liquid' simulation LW and SW CRE by cloud (supercooled) liquid water was excluded to distinguish between the ice-only and liquid-only radiative impacts. As we focus solely on the radiative effect of clouds on the SMB, their role as precipitation source is not considered.

These simulations were performed using a physically based broadband albedo parameterization^53^,^60^, which was implemented in SNOWPACK and accounts for albedo variations in function of the specific surface area of the snow/ice profile, cloud optical depth, SZA and concentration of light-absorbing carbon in the snow/ice, but with a lower limit of albedo values that corresponds to the spatially variable ice-albedo values derived from MODIS^53^. The specific surface area of the snow/ice profile was derived from SNOWPACK geometric grain size per layer, whereas the cloud optical depth was calculated from the hybrid satellite-climate model LWP/IWP data set. Finally, the concentration of light-absorbing carbon in the snow/ice was set to a fixed value per layer together with the other SNOWPACK model parameters (Supplementary Table 2). SNOWPACK was run in an offline configuration, that is, identical atmospheric temperature, wind speed, relative humidity and precipitation were used for all three simulations. This implies that we do not allow the atmosphere to react to changes in the snowpack.

The performance of SNOWPACK to simulate GrIS surface conditions was assessed based on the comparison of SNOWPACK SMB with RACMO SMB and stake measurements, SNOWPACK melt with satellite-observed melt and SNOWPACK refreezing rates with refreezing rates from literature. First, the yearly average SMB for each grid box was calculated, showing a close correspondence between SNOWPACK and RACMO SMB (Supplementary Fig. 9). We would like to stress here that this comparison is not aimed at an absolute evaluation of GrIS SMB estimates, but merely serves as a check that SNOWPACK simulates a similar SMB when forced by the same meteorological inputs as RACMO2.3. Integrated over the GrIS, this is also confirmed by the strong agreement between the red and black curves in Fig. 3a. The limited amount of SMB observations through yearly stake measurements hinder a Greenland-wide observation-based evaluation of SMB. Yet, we compared the SNOWPACK-simulated SMB with observations from the K-transect^62^ in the same period. The spatial resolution of the SNOWPACK runs prevents individual comparisons for all stations of the K-transect. However, the S10 station is representative for an accumulation pixel, whereas the other stations cover an ablation pixel. The yearly mean recorded SMB at S10 is 203 kg m^−2^, whereas SNOWPACK indicates 243 kg m^−2^. The other stations have a mean yearly SMB of −1,714 kg m^−2^, whereas the corresponding grid box of SNOWPACK has an SMB of −1,708 kg m^−2^. This comparison gives a good indication that the SNOWPACK estimates are sufficiently in line with the available SMB observations in the field. In addition, melt occurrence was calculated, to assess the ability of SNOWPACK to reproduce observed melt events from spaceborne passive microwave data^63^. In this framework, melt days in the SNOWPACK simulations were defined as days during which hourly surface temperatures reached the melting point within the window of overpass of the satellite^63^ to detect surface melt. The original daily melt detections^63^ were aggregated to the 2° by 2° grid for comparison with SNOWPACK. The yearly amount of melt days for each grid box agree reasonably well between observations and SNOWPACK simulations (Supplementary Fig. 10). Some scatter exists due to the definition of melt days, slight timing offsets and relatively coarse resolution of the SNOWPACK simulations. Nonetheless, the general agreement with observations is good and also the spatial patterns are captured well (Supplementary Fig. 11). Based on our definition of melt days, we find a mean yearly melt index (number of days melt is observed, multiplied by the area where the melt occurs) of 3.45 × 10^7^ km^2^ days, whereas the melt index calculated from the observed melt data^63^ is 3.60 × 10^7^ km^2^ days, a difference of <5%. In addition, also the timing of simulated melt events was compared with observed melt events, using the same data set^63^. The result shows a very good agreement between both simulated SNOWPACK melt events and observed melt events (Supplementary Fig. 12). Finally, we quantified the mean refreezing rate in SNOWPACK in the all-sky simulation as 45%, whereas another study^64^ found 42±4 %. The SMB, melt and refreezing rate comparisons illustrate the good performance of SNOWPACK for simulating the GrIS SMB.

### Uncertainty derivations

Radiative fluxes retrieved from satellite observations carry an inherent uncertainty due to many different factors. In the retrieval of CRE from satellite observations, we have accounted for the following sources of uncertainty: (i) LW/SW radiative flux retrievals from 2B-FLXHR-LIDAR, (ii) cloud enhancement factors *F* predicted by the neural networks and (iii) assumed surface albedo values in 2B-FLXHR-LIDAR. To incorporate uncertainty estimates based on the LW/SW radiative flux retrievals by 2B-FLXHR-LIDAR, we used the comparisons with ground-based radiative flux measurements from AWS observations. In this framework, a general RMSE value for both LW fluxes (9.3 W m^−2^) and SW fluxes (22.5 W m^−2^) was derived from the individual comparisons with the stations. These RMSE values represent the mean uncertainty of the satellite-retrieved radiative fluxes over the GrIS and were subsequently used to perturb the obtained satellite LW/SW fluxes (100 iterations), by introducing random errors from a normal distribution *N*(*μ*=0; *σ*=RMSE). These were then used as inputs to the neural networks, where an additional uncertainty was added by perturbing the predicted cloud enhancement factors *F* using random errors from a normal distribution with means and s.d. based on the network performance results (Supplementary Fig. 4). Finally, a last source of uncertainty was added to the 100 sets of radiative fluxes, based on the surface albedo assumption. Comparison of the albedo values used in the 2B-FLXHR-LIDAR algorithm with the physically based albedo retrievals in the RACMO model resulted in a mean bias of 0.04±0.05. A random error was introduced based on these characteristics, from which updated radiative fluxes were calculated. The final 100 sets of radiative fluxes were used to calculate corresponding sets of CRE and include the three most important sources of uncertainty. The s.d. of this data set provides the combined uncertainty estimate of CRE values that are reported in this study.

SMB terms in SNOWPACK contain a combination of uncertainties that accumulated in earlier steps of our methodology. These uncertainties were estimated using a combination of (i) perturbed CRE to force SNOWPACK and (ii) inherent SNOWPACK model uncertainty. As the SMB terms for the different simulations depend strongly on the microhpysical characteristics of clouds, we quantified the LWP/IWP uncertainty of the hybrid satellite-climate model data set by comparing these values with the satellite observations. We found a mean LWP uncertainty of 35% and IWP uncertainty of 12%. Therefore, we performed four additional SNOWPACK runs using all possible combinations of adding/removing 35% of the LWP and 12% of the IWP at each timestep, and calculating the corresponding radiative fluxes. The range of resulting outputs for the all-sky and no-liquid simulations were used as uncertainty due to the amount of cloudiness in the SNOWPACK runs. Inherent SNOWPACK uncertainty was estimated by comparing SNOWPACK and RACMO estimates of the SMB terms (Fig. 3a). Results of this comparison showed that the uncertainty on the total GrIS SMB is ∼10%, whereas the uncertainty on the individual components is ∼50% for sublimation and ∼20% for runoff. Finally, both sources of uncertainty (inherent model uncertainty and cloudiness) on SNOWPACK SMB and individual mass terms were combined (whiskers and shaded areas in Fig. 3, and all mass-related uncertainties in the text).

### Code availability

All codes that have contributed to the results reported in this study are available on request. The 2B-FLXHR-LIDAR data are available at http://www.cloudsat.cira.colostate.edu/.

## Additional information

**How to cite this article:** Van Tricht, K. *et al*. Clouds enhance Greenland ice sheet meltwater runoff. *Nat. Commun.* 7:10266 doi: 10.1038/ncomms10266 (2016).

## Supplementary Material

Supplementary Information Supplementary Figures 1-12, Supplementary Tables 1-2, Supplementary Methods and Supplementary References

## Figures and Tables

**Table 1:** Seasonal and yearly cloud frequencies and mean water paths.

	**All clouds (%)**	**Ice-only (%)**	**Liquid-bearing (%)**	**Mean IWP (g** **m**^−2^**)**	**Mean LWP (g** **m**^−2^**)**
Spring (MAM)	63	41	22	20	6
Summer (JJA)	66	20	46	19	16
Autumn (SON)	68	41	27	25	8
Winter (DJF)	70	55	15	23	5
Year	67	39	28	21	9

GrIS, Greenland ice sheet; IWP, ice-water path; LWP, liquid-water path. MAM, March-April-May; JJA, June-July-August; SON, September-October-November; DJF, December-January-February.

The observed total cloud frequency (in %) over the GrIS (2007–2010) is fairly constant year round, but the partitioning and water paths of ice and liquid water exhibit strong seasonal variations. It is noteworthy that in the event of multiple detected cloud layers in a vertical profile, these are combined and reported as a single cloudy profile in these statistics.
