# Supplementary material for: Clouds enhance Greenland ice sheet meltwater runoff
Source: Nat Commun. 2016 Jan 12;7:10266. doi: 10.1038/ncomms10266 (PMC4729937; doi:10.1038/ncomms10266)
Supplement: Supplementary Information — Supplementary Figures 1-12, Supplementary Tables 1-2, Supplementary Methods and Supplementary References [file ncomms10266-s1.pdf]

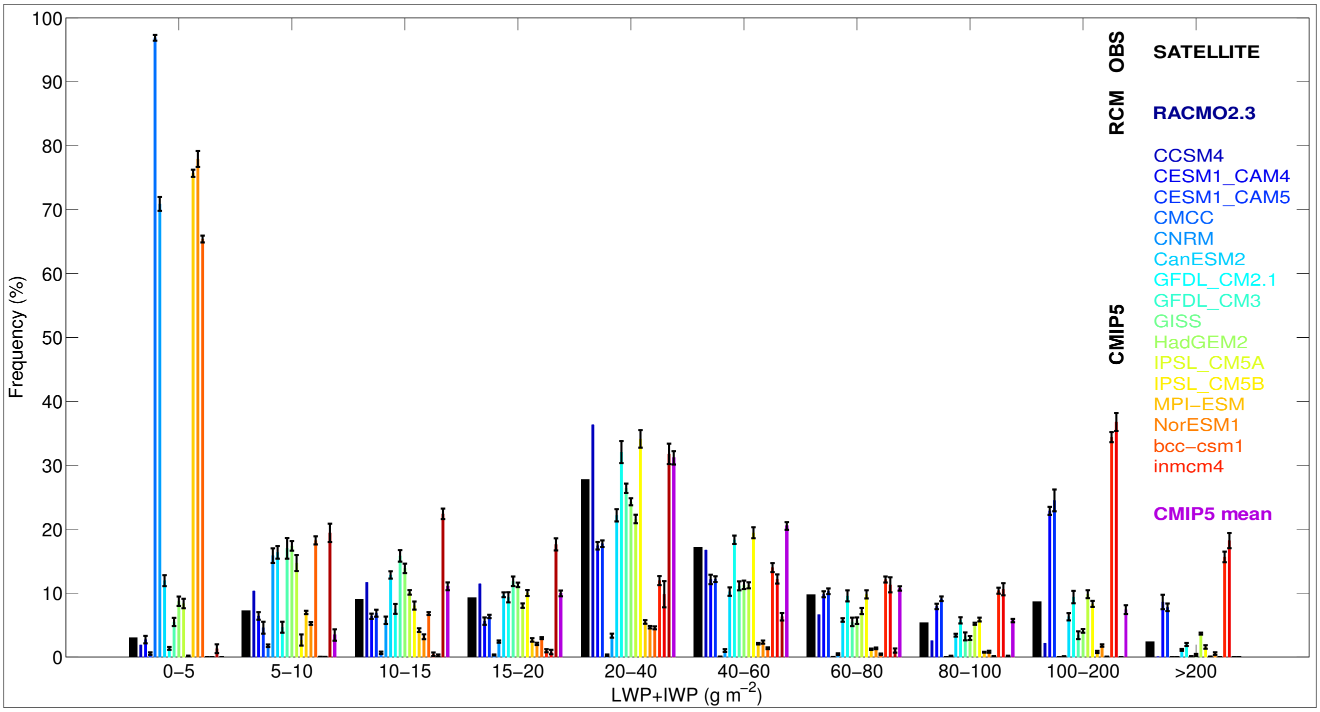

Supplementary Figure 1 | Comparison of cloud frequency over the GrIS between climate models and satellite observations. CMIP5 and RACMO2.3 inter-model spread of IWP+LWP (g m^{-2}) shows the large variability in simulated clouds between the different models. None of the models is able to represent the satellite-observed spectrum of LWP + IWP (black bars). CMIP5 whiskers indicate intra-model variability of cloudiness in four consecutive years over the 1986-2005 period, which is much lower than the observed inter-model spread.

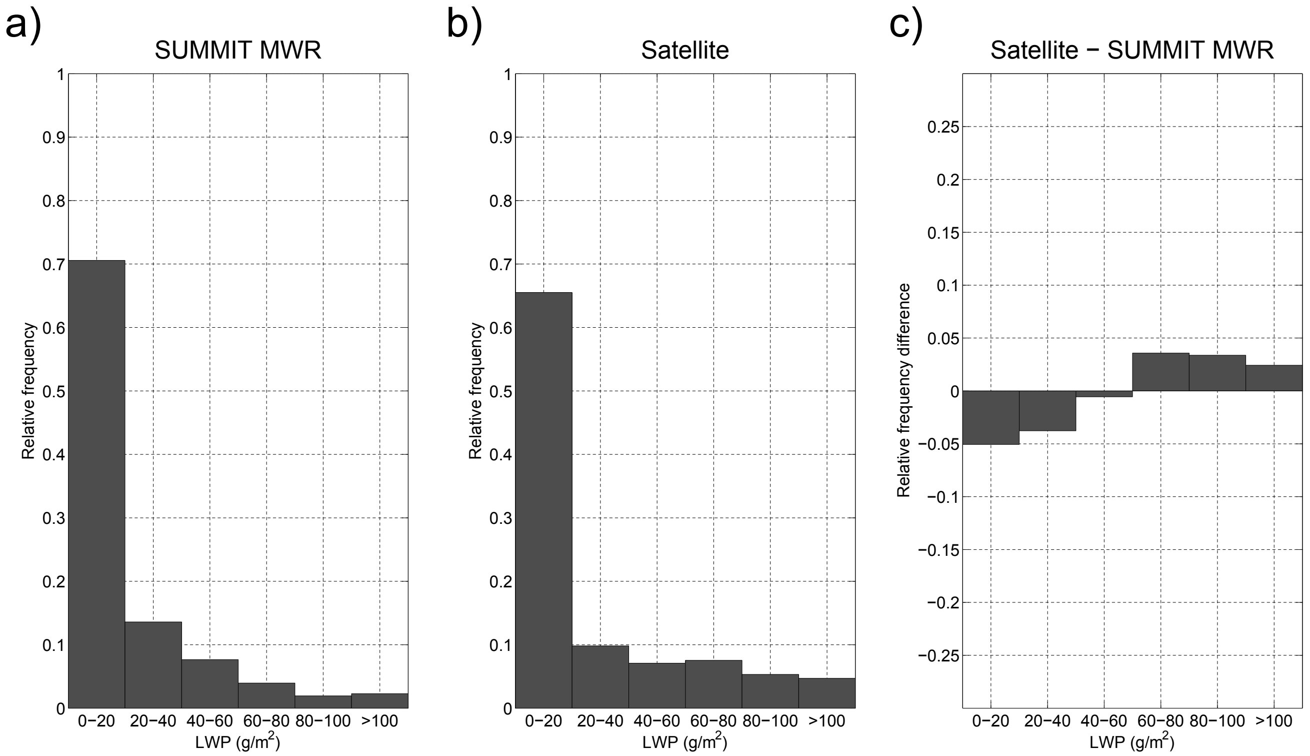

Supplementary Figure 2 | Comparison of LWP relative frequency histograms at Summit (Jul-Dec 2010). **(a)** Retrieved by the ground-based MWR at Summit station. **(b)** Retrieved by satellite cloud observations (refined 2B-FLXHR-LIDAR product). **(c)** The difference histogram shows the strong agreement between satellites and ground-based observations.

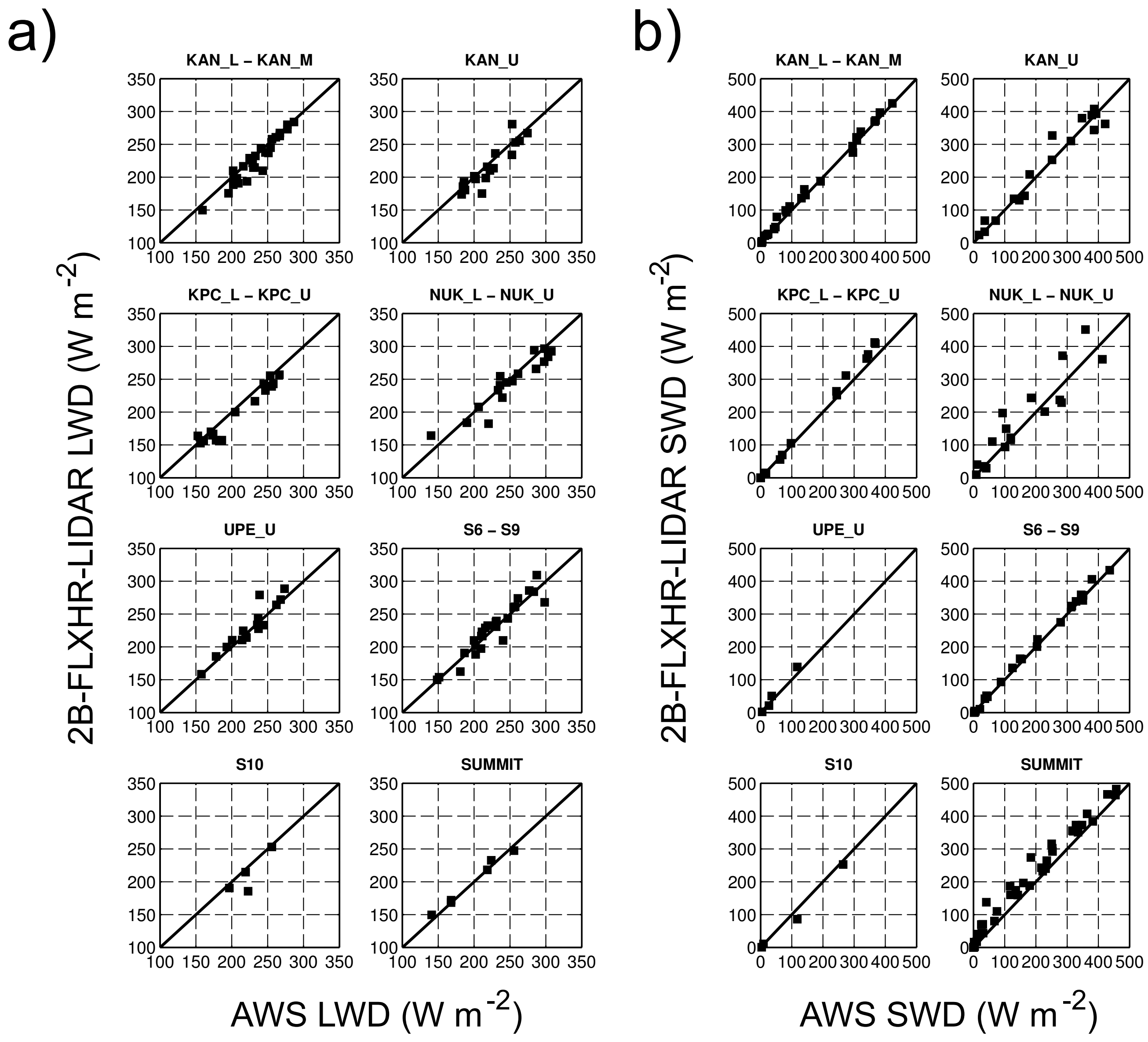

Supplementary Figure 3 | Comparison of monthly mean radiative fluxes bewteen satellite retrievels and AWS observations. Comparison of **(a)** mean downwelling LW (LWD) and **(b)** mean downwelling SW (SWD) retrievals (2007-2010) over 8 different locations and a total of 11 AWS stations shows the close agreement between satellite radiative flux retrievals (refined 2B-FLXHR-LIDAR product) and ground-based observations.

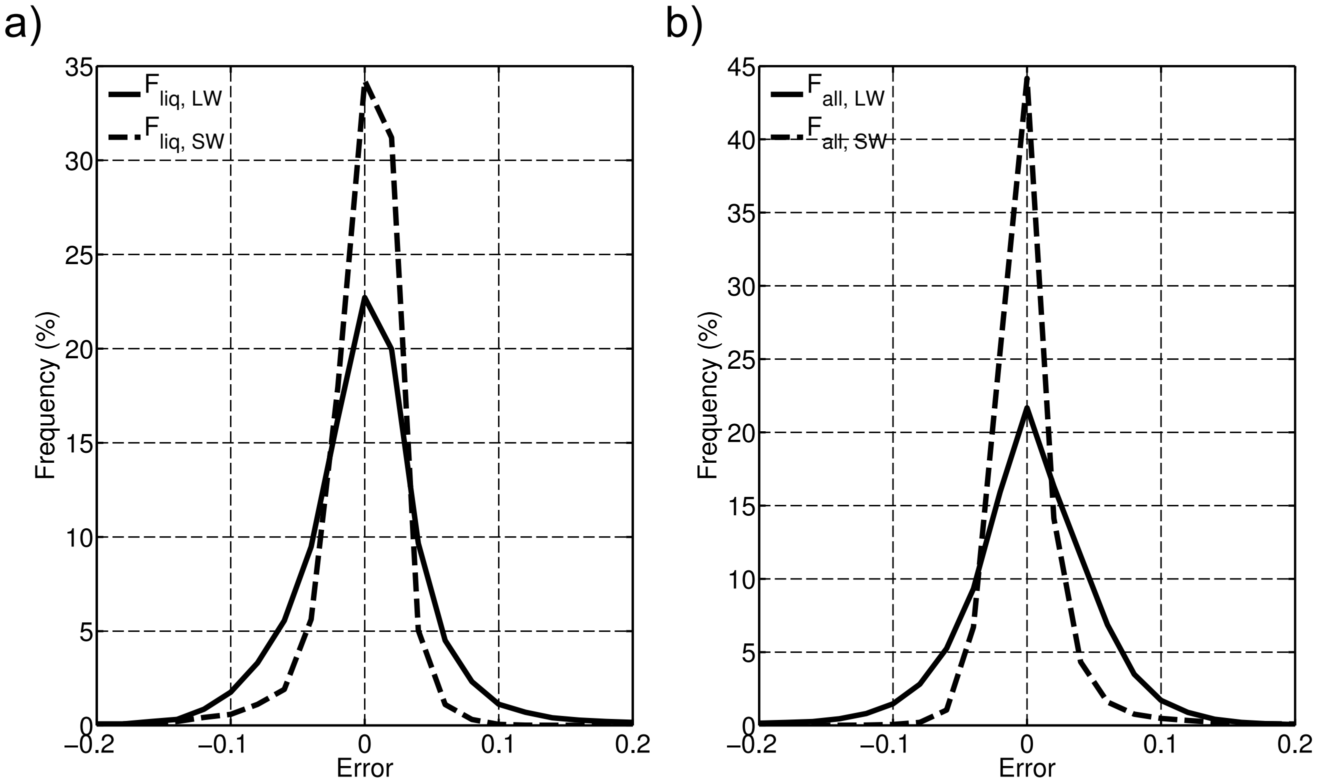

Supplementary Figure 4 | Performance of the neural networks. Error histograms of **(a)** $F_{\mathrm{liq,\ LW}}$ and $F_{\mathrm{liq,\ SW}}$ factors, and **(b)** $F_{\mathrm{all,\ LW}}$ and $F_{\mathrm{all,\ SW}}$ factors, as predicted by neural networks vs. calculated factors from the RTM runs.

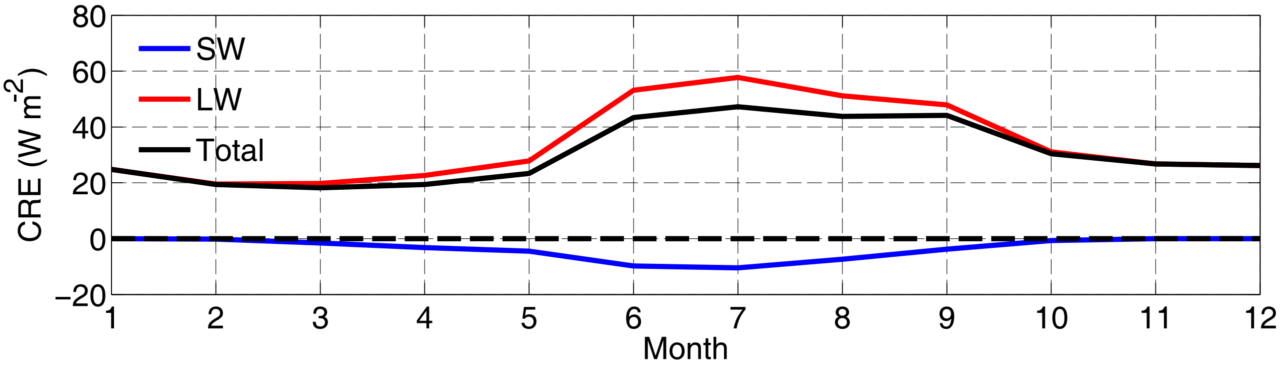

Supplementary Figure 5 | Seasonal cloud radiative effect at Summit. Monthly mean LW, SW and total net CRE at Summit, Greenland (2007-2010), as derived from the observational-driven neural networks.

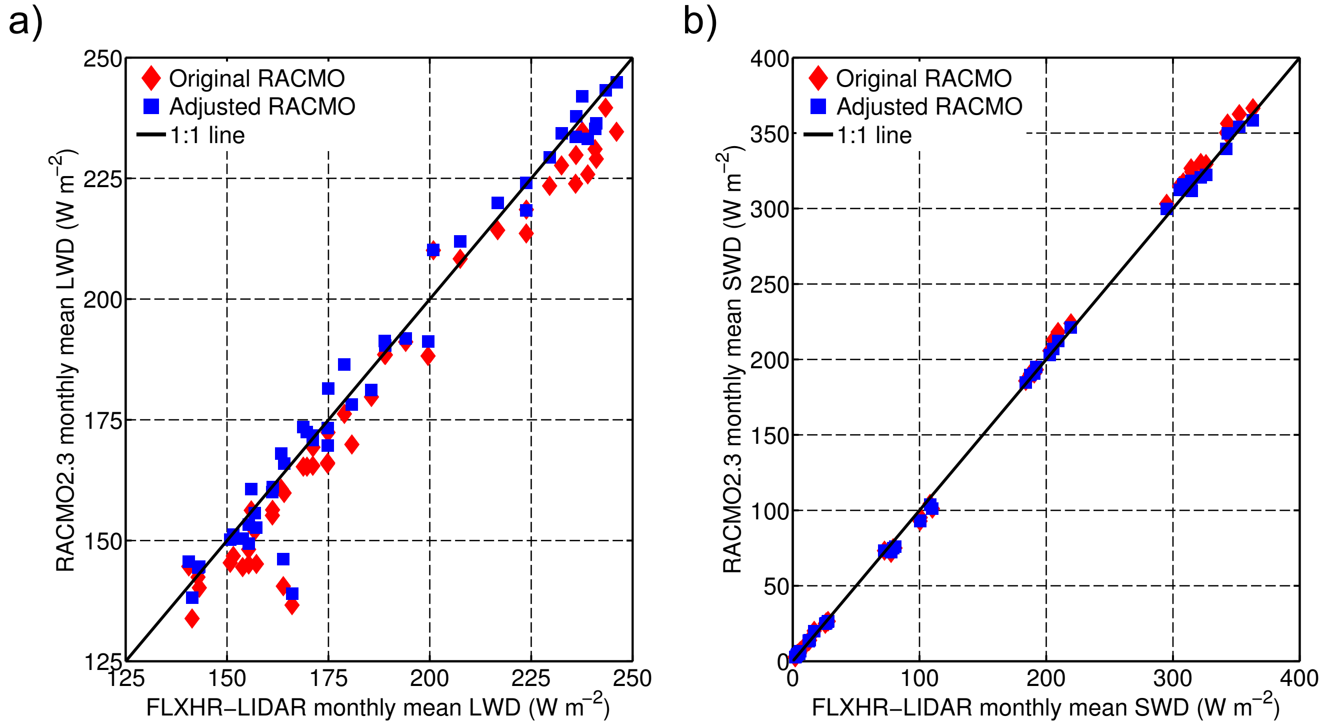

Supplementary Figure 6 | Radiative flux comparisons between satellite observations and the regional climate model RACMO2.3. Comparison of monthly mean **(a)** LWD and **(b)** SWD fluxes between (red diamonds) original RACMO2.3 radiative fluxes and 2B-FLXHR-LIDAR satellite retrievals, and (blue squares) adjusted RACMO2.3 radiative fluxes and 2B-FLXHR-LIDAR satellite retrievals. All values have been averaged over the entire GrIS domain.

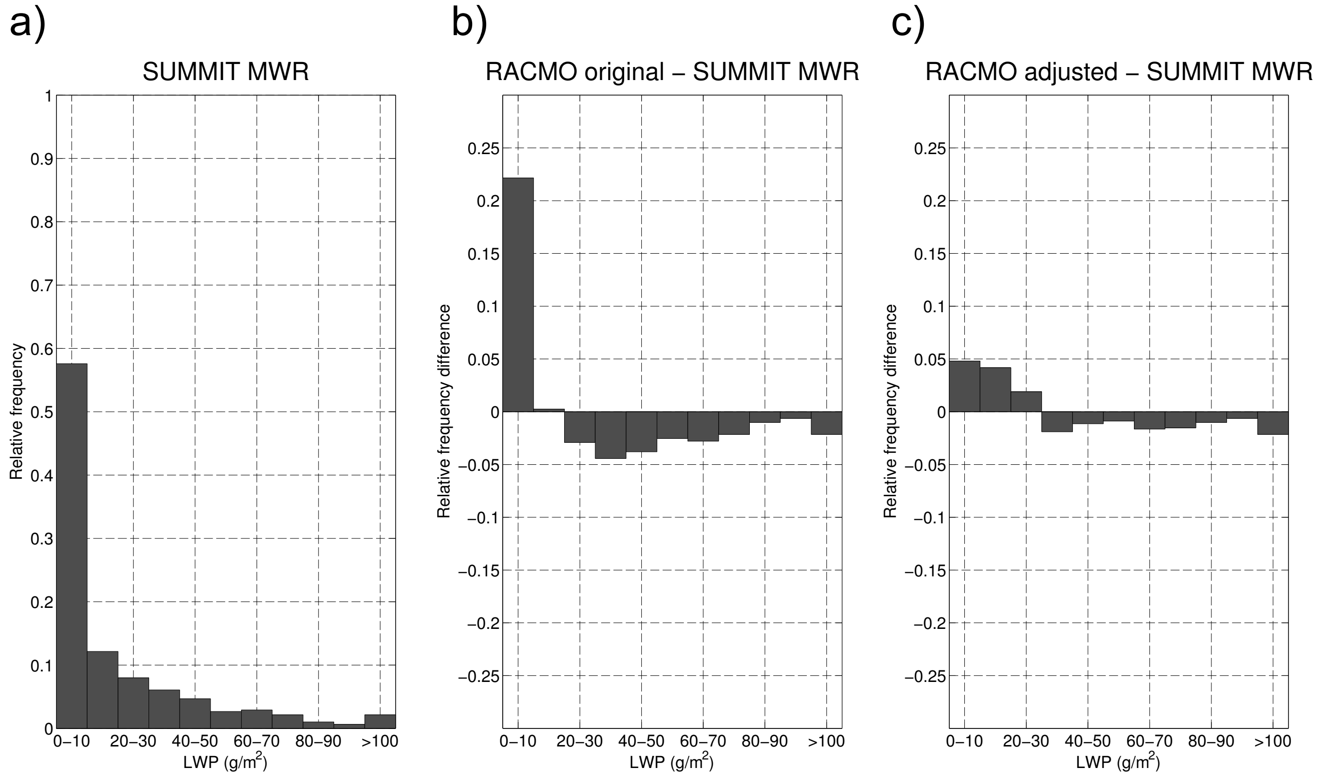

Supplementary Figure 7 | LWP histogram comparisons. **(a)** Relative frequency histogram of ground-based retrievals of LWP at Summit by MWR and frequency difference histograms of **(b)** original model and **(c)** hybrid satellite-climate model LWP time series vs. ground-based retrievals. The MWR retrievals were averaged to match the climate model temporal resolution.

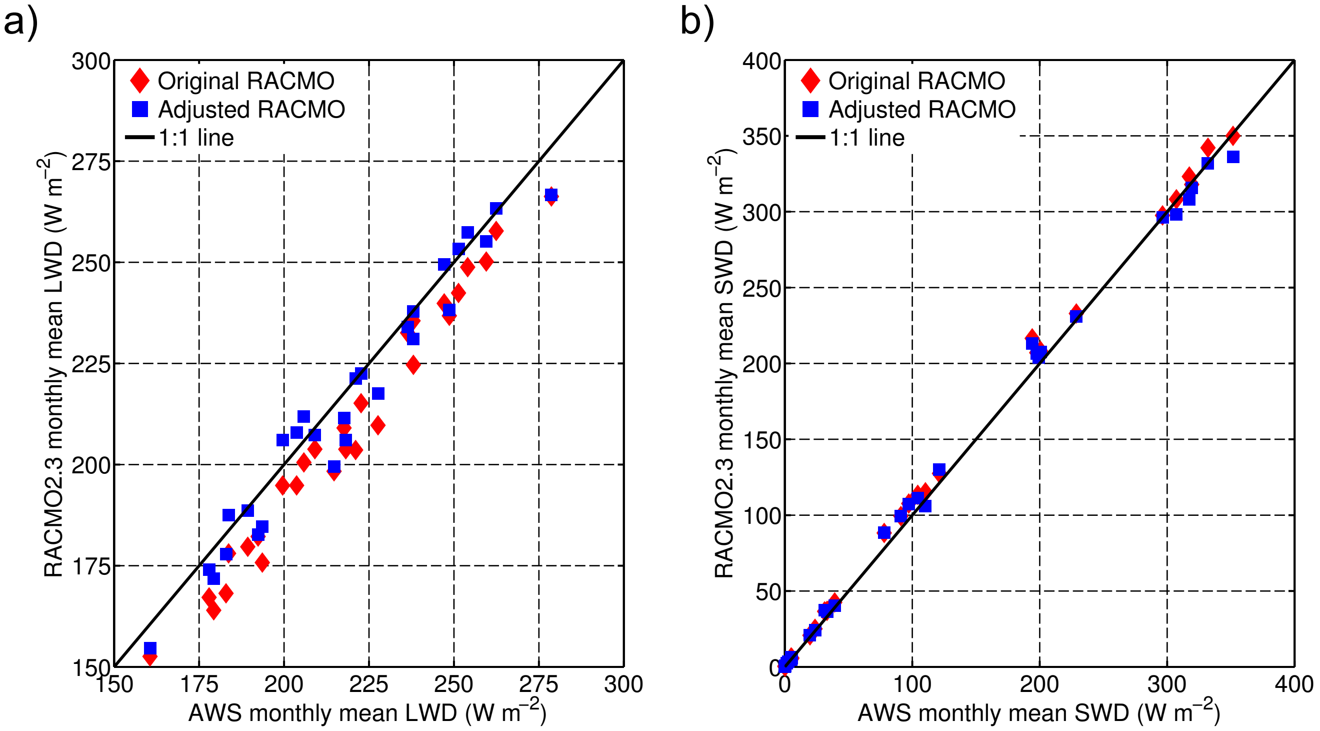

Supplementary Figure 8 | Comparison of radiative fluxes between climate model and AWS observations. Comparison of **(a)** downwelling LW (LWD) and **(b)** downwelling SW (SWD) fluxes measured by AWS stations to modelled fluxes before and after adjusting to the hybrid LWP/IWP time series.

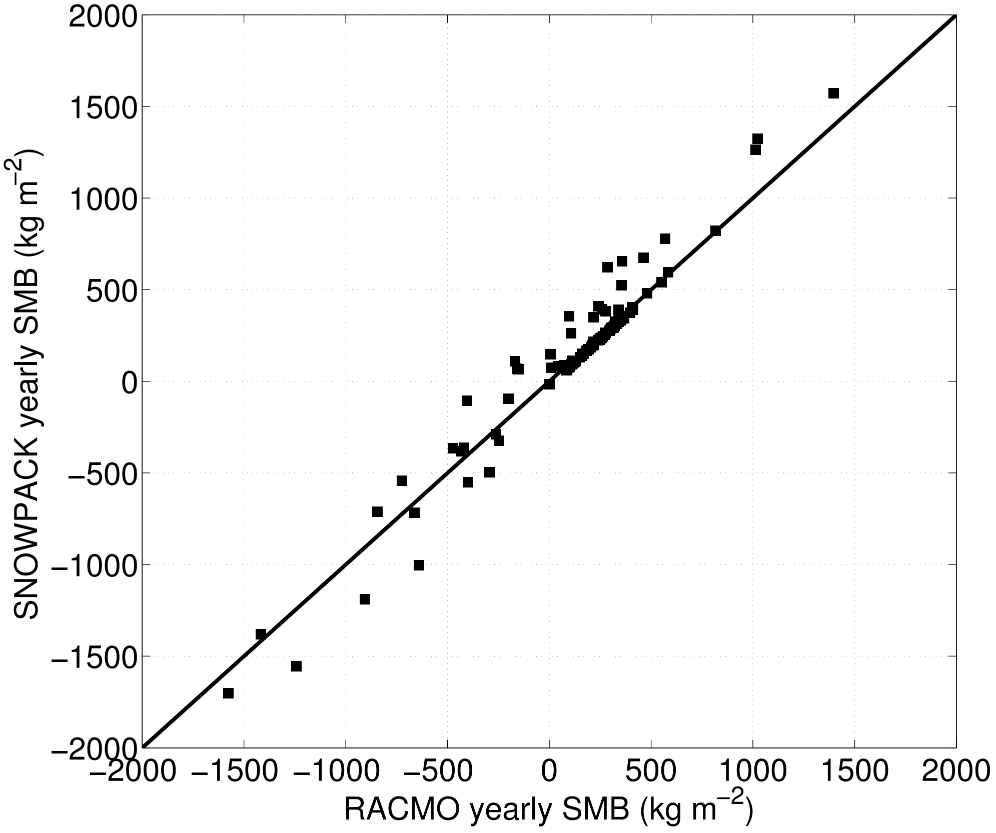

Supplementary Figure 9 | Yearly average SMB in each gridbox for RACMO and SNOWPACK show a close correspondence.

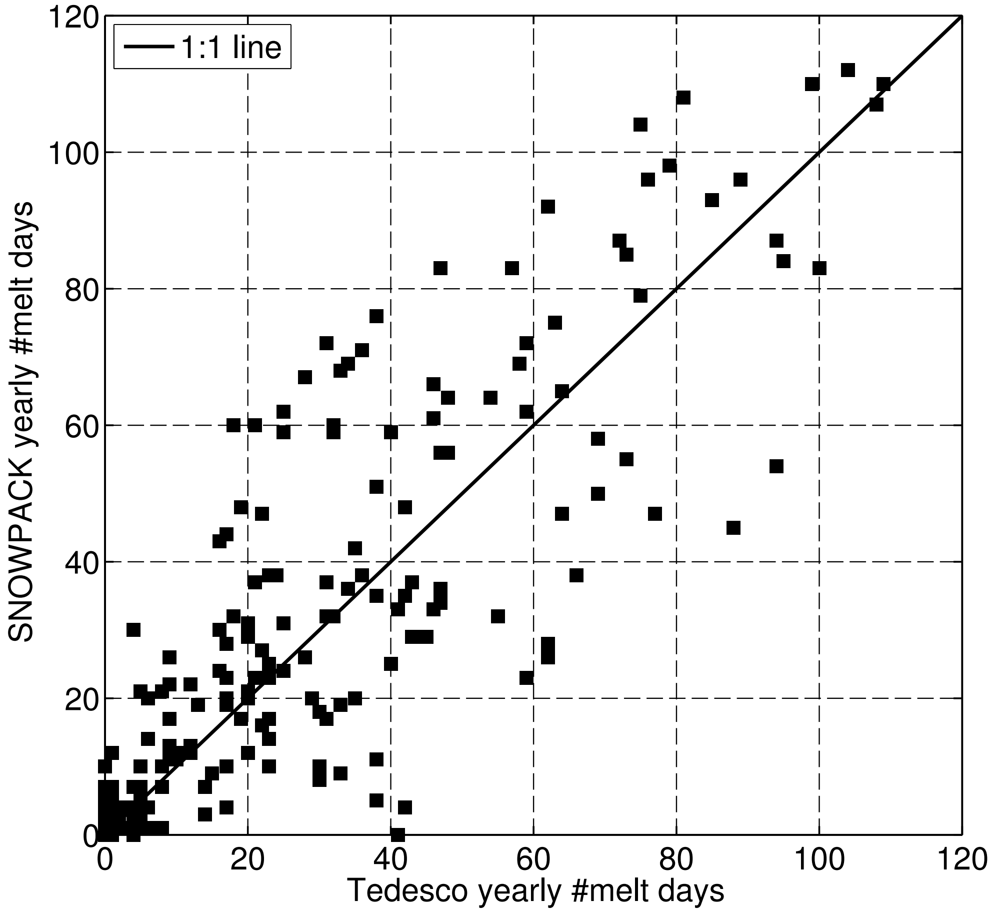

Supplementary Figure 10 | Comparison of yearly number of melt days as simulated by SNOWPACK and observed by satellite imagery.[1]

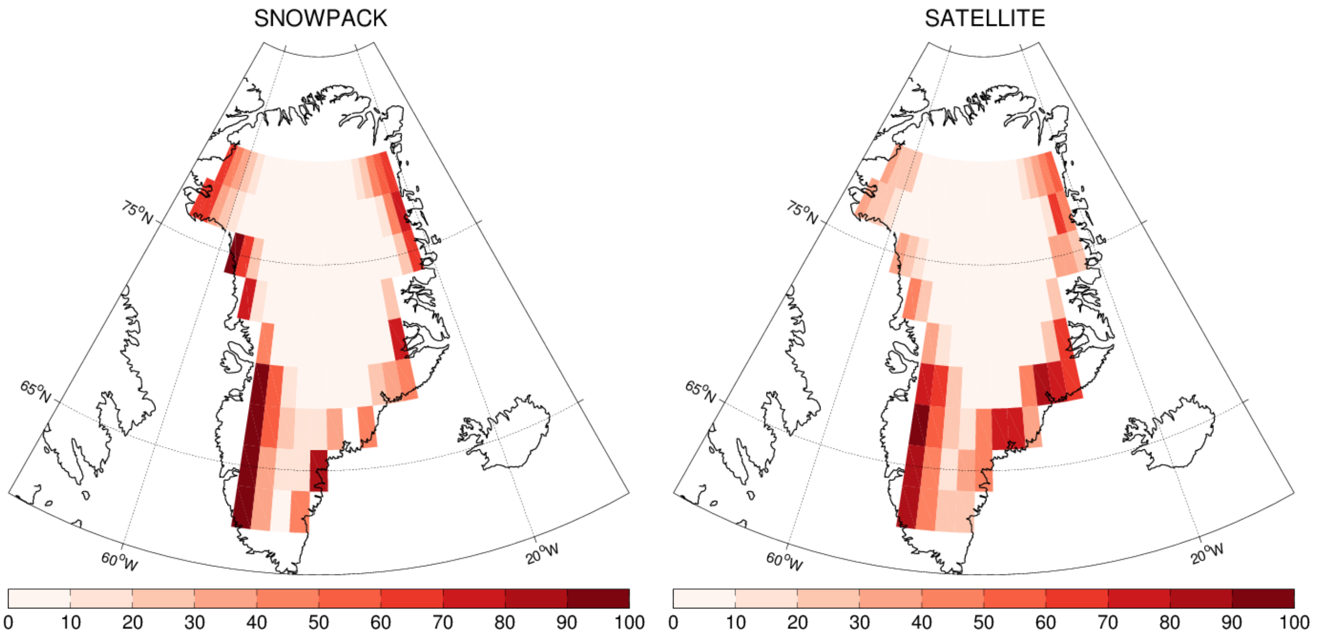

Supplementary Figure 11 | Spatial patterns of average yearly number of melt days agree well between SNOWPACK simulations and satellite observations.

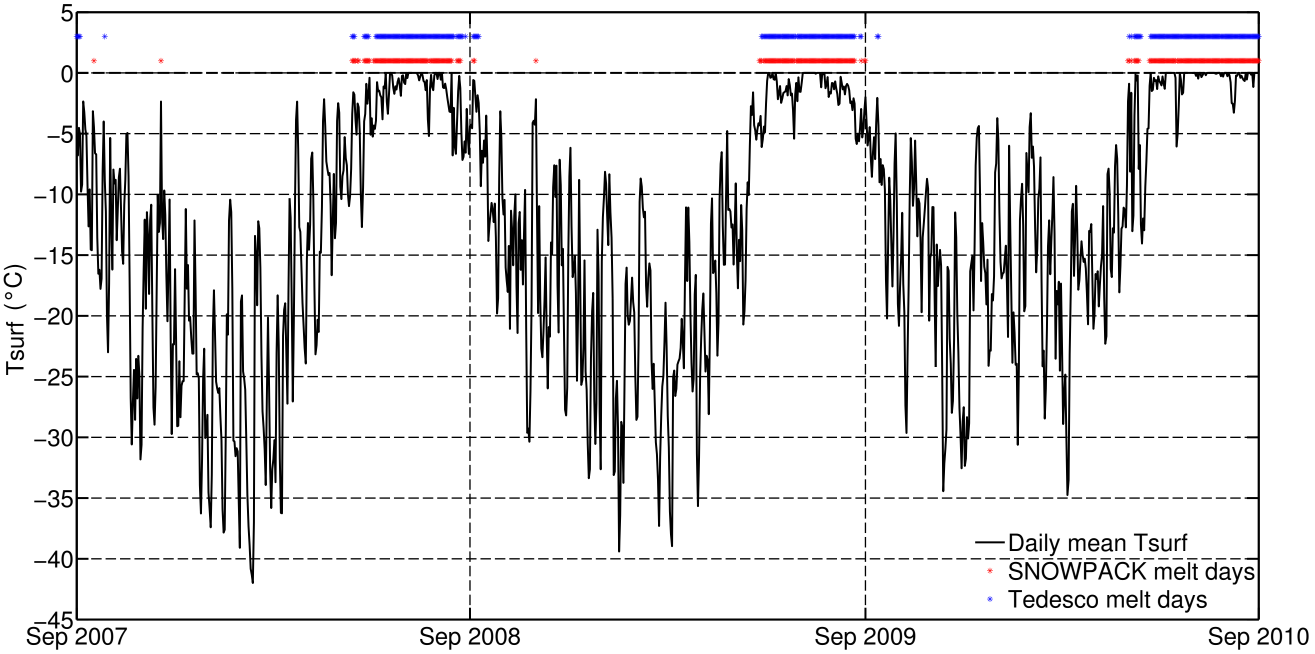

Supplementary Figure 12 | Time series of daily surface temperature and comparison of melt days between simulations and observations. Daily average simulated surface temperature (Tsurf) by SNOWPACK (Sep 2007 - Sep 2010) with simulated melt days in red and observed melt days in blue,[1] for the ablation gridcell with center coordinates 69N-49E. A melt day in SNOWPACK is defined as a day during which hourly surface temperatures reach the melting point for at least 6 hours.

Supplementary Table 1 | Mean bias and RMSE (W m^{-2}) between 2B-FLXHR-LIDAR and AWS observations, based on monthly mean values from 2007-2010.

	LWD		SWD	
**AWS ID**	**Bias**	**RMSE**	**Bias**	**RMSE**
KAN_L - KAN_M	-7.4	12.3	8.0	18.6
KAN_U	-5.3	13.8	1.6	30.0
KPC_L - KPC-U	-8.7	12.6	11.8	20.5
NUK_L - NUK-U	-5.3	16.0	16.0	50.7
UPE-U	3.9	12.4	6.6	13.3
S6 - S9	1.1	12.9	4.9	9.7
S10	-12.7	19.0	-11.4	17.2
SUMMIT	2.2	6.2	23.4	33.6
**Average**	**-4.0**	**13.2**	**7.6**	**24.2**

Supplementary Table 2 | SNOWPACK setup parameters

Parameter	Setup
Modelling time step	60 min
Atmospheric stability correction model	Monin-Obukhov[2]
New snow density model	Lehning new[3]
Albedo parameterization	Gardner & Sharp[4]
Water transport model	bucket[5]
SW radiation penetration	Multi-band[3]
Constant geothermal heat flux	0 W m^{-2}
Aerodynamic roughness length	0.001 m
Temperature threshold for rain/snow transition	1.2° C
Initial snow grain radius (geometric)	55 μm
New/minimum element size	0.01 m
Soot concentration	0.2 ppmv
Superimposed ice	No
Drifting snow	No
Spinup period	1 Sep 1996 - 1 Sep 2007
Spinup initial snowpack thickness	335 m (200x1 m + 300x0.25 m + 600x0.1 m)
Spinup initial snow temperature	1996-2010 mean surface temperature

Supplementary Methods

Clouds over Greenland in climate models

To study the representation of clouds and their ice and liquid water contents in state-of-the-art climate models, monthly mean LWP and IWP amounts were acquired from the RCM RACMO2.3 and from the available CMIP5 models ('clwvi' variable in CMIP5 archive). For RACMO2.3, the period 2007-2010 was used to agree with the satellite observations. For CMIP5, we did not select the 2007-2010 period, because (i) the historical CMIP5 simulations end in 2005, and (ii) CMIP5 climate models are not forced by observed meteorology, limiting the direct comparison with observations; instead, we selected the last 20 years of the CMIP5 historical simulations (1986-2005). Cloud data from 28 CMIP5 historical model simulations could be retrieved, but some of these use the same parent model with slightly differing setup or complexity; for the remainder of the CMIP5 ensemble, no clwvi data were available. The data from the simulations with the same model were averaged, leaving 16 main models (CCSM4, CESM1_CAM4, CESM1_CAM5, CMCC, CNRM, CanESM2, GFDL_CM2.1, GFDL_CM3, GISS, HadGEM2, IPSL_CM5A, IPSL_CM5B, MPI-ESM, NorESM1, bcc-csm1 and inmcm4, see Supplementary Fig. 1) for this study. All model data were interpolated on the 2° by 2° grid used for the satellite data over the Greenland ice sheet (GrIS), and classified according to their monthly mean LWP + IWP amounts. The same exercise was repeated for the satellite data (see Methods in main paper). To verify that a direct comparison between four years of satellite data and four different years of CMIP5 model data is justified, we checked the variability in LWP + IWP amounts in each CMIP5 member for each possible combination of monthly mean values in four consecutive years (ranging between 1986-2005). The whiskers in Supplementary Fig. 1 for the CMIP5 models show that the variability within the 1986-2005 period for each CMIP5 member is much smaller than the inter-model variability, justifying a qualitative comparison of the frequency histograms. Supplementary Fig. 1 shows that LWP + IWP values vary substantially among the available CMIP5 models and RACMO2.3 RCM. For instance, LWP + IWP values between 0 and 5 g m^{-2} occur between 0 and 97% according to the models, whereas the satellite indicates a frequency of 3%. Intermediate LWP + IWP values between 20 and 40 g m^{-2} occur around 28% of the time according to the observations, whereas the model frequencies range between 0% and 36%. High LWP + IWP values >100 g m^{-2} occur 11% of time according to satellite observations, while 5 out of 17 models have occurrences below 1%. None of the 16 CMIP5 models or the RCM is able to represent the observed spectrum of LWP + IWP values, with some of them having too low water paths and others having too high water paths, although the CMIP5 multi-model mean (calculated from the average monthly LWP + IWP values in each gridbox over all CMIP5 members) is closer to the observed distribution. The inter-model spread of LWP + IWP signals that there is a substantial uncertainty in assessing the impact of clouds on the GrIS using individual state-of-the-art climate models. Moreover, an even higher spread exists in the liquid-ice partitioning of clouds (not shown), with a tendency of the models to overestimate the ice fraction at the expense of (supercooled) liquid water. This highlights the need

for an observation-based assessment of cloud impacts over the GrIS.

Supplementary references

[1] Tedesco, M. Greenland Daily Surface Melt 25km EASE-Grid, 2007-2010. *New York, NY, USA: City College of New York, The City University of New York. Digital media.* (2014).

[2] Monin, A. & Obukhov, A. Basic laws of turbulent mixing in the surface layer of the atmosphere. *Contrib. Geophys. Inst. Acad. Sci. USSR* (1954).

[3] Lehning, M., Bartelt, P., Brown, B., Fierz, C. & Satyawali, P. A physical SNOWPACK model for the Swiss avalanche warning: Part II. Snow microstructure. *Cold Regions Science and Technology* **35**, 147–167 (2002).

[4] Gardner, A. S. & Sharp, M. J. A review of snow and ice albedo and the development of a new physically based broadband albedo parameterization. *Journal of Geophysical Research* **115**, 1–15 (2010).

[5] Wever, N., Fierz, C., Mitterer, C., Hirashima, H. & Lehning, M. Solving Richards Equation for snow improves snowpack meltwater runoff estimations in detailed multi-layer snowpack model. *The Cryosphere* **8**, 257–274 (2014).
